# Enhanced tantalum and niobium recovery from fine-grained low-grade Abu Dabbab ore using Falcon concentration and magnetic separation

**DOI:** 10.1038/s41598-025-92819-7

**Published:** 2025-03-26

**Authors:** El-Sayed R. E. Hassan, Yun Chen, N. A. Abdel-Khalek, A. M. Elbendari

**Affiliations:** 1 Minerals Beneficiation and Agglomeration Department, Minerals Technology Institute, Central Metallurgical Research & Development Institute (CMRDI), Helwan, P.O. Box 87, Cairo, 11722 Egypt; 2 School of Materials and Chemical Engineering, Hunan Institute of Engineering, Xiangtan, China

**Keywords:** Tantalum oxide, Niobium oxide, Abu Dabbab locality, Falcon concentrator, Box-Mag magnetic separator, Engineering, Materials science

## Abstract

Tantalum (Ta) and niobium (Nb) possess unique properties that make them invaluable across various industries. However, their similar chemical characteristics and natural co-occurrence challenge their separation. This study aims to optimize the recovery of Ta and Nb from fine-grained, low-grade Abu Dabbab tantalite ore (Egypt) using integrated Falcon centrifugation and magnetic separation. The beneficiation process commenced with gravity separation via a Falcon SB40 concentrator, which was optimized through a Box-Behnken design to assess the feed rate, fluidization water, and centrifugal field effects. Under optimal conditions (89.30 g/min feed rate, 4.40 psi fluidization water, 200 G centrifugal field), a concentrate grade of 2.00% Ta_2_O_5_ and 0.705% Nb_2_O_5_ was achieved, with recoveries of 85.70 and 84.35%, respectively. Subsequent magnetic separation using a Box-Mag Rapid LHW separator (2.0 T field intensity, 21.05% matrix loading, 11.40% pulp density) further enriched the concentrate to 6.22% Ta_2_O_5_ and 2.24% Nb_2_O_5_, with recoveries exceeding 94%. Cassiterite (SnO_2_) was co-enriched to 17.50% (195-fold enrichment), while the albite and mica fractions were recovered as by-products. The characterization study confirmed the efficacy of the sequential process, yielding a high-grade concentrate. The combined approach demonstrates a scalable, efficient pathway for upgrading fine-grained, low-grade tantalite ores, achieving 250-fold enrichment ratios for Ta and Nb. This work underscores the viability of integrating optimized gravity and magnetic separation for the sustainable recovery of Ta and Nb critical metals, offering significant economic and technological benefits for resource utilization from complex ores.

## Introduction

Egypt possesses significant mineral resources that can potentially fulfill part of the industrial demand. Among these resources, tantalum and niobium stand out as transition metals with very similar physical and chemical properties, which is why they are often grouped together^1,2^. Tantalum and niobium exhibit distinct characteristics, including high melting points and exceptional resistance to acid corrosion. They are ductile metals that can be easily fabricated and are effective conductors of heat and electricity. These unique characteristics make tantalum and niobium valuable for various industrial applications. Tantalum is used in the manufacture of surgical instruments, reaction vessels, alloys, and wires, as well as in lenses, electron tubes, and watches. Tantalum pentoxide (Ta_2_O_5_) is employed in producing of advanced materials, such as capacitors for cell phones and laptops^1–4^. Niobium finds application in jewelry, capacitors, superconducting magnets, optical lenses, and medical devices. It is also used in Fe-, Co-, and Ni-based superalloys for jet engines, in nuclear applications. Niobium oxide is utilized in the production of lithium niobate for cell phones^3–5^.

The primary sources of tantalum and niobium are minerals such as tantalite, columbite, pyrochlore, and coltan (columbite-tantalite)^4,6^. The largest high-grade deposits are located in Canada, Brazil, Nigeria, Zaire, and Russia. In Egypt, tantalum and niobium mineralization is found in hard rock deposits and placers in the Southeastern Desert. The Geological Survey of Egypt has identified the presence of niobium and tantalum-bearing apogranites in Abu Dabbab and Nuweibi. The Abu Dabbab deposit contains a total of 45 million tons of ore, with concentrations of 0.025% Ta_2_O_5_, 0.001% Nb_2_O_5_, and 0.09% Sn. This deposit is primarily a tantalite deposit, with a Ta_2_O_5_ ratio of approximately 3:1. The apogranites in this region are predominantly made up of feldspars (primarily albite), quartz, muscovite, and biotite^6^.

The beneficiation of niobium and tantalum can be achieved through physical and/or chemical techniques. Given the low concentrations of these metals, the most cost-effective approach involves gravity separation. The most commonly used gravity concentrators are shaking tables and centrifugal concentrators. Magnetic and electrostatic separation methods have been employed to upgrade niobium and tantalum minerals^1,7^.

Ghorbani et al. (2017) enhanced tantalite from the Penouta mine (Spain) by utilizing shaking tables and Knelson concentrators, achieving an enrichment ratio of approximately 80 times for Ta_2_O_5_ and 45 times for Nb_2_O_5_^8^. Yuan et al. (2015) employed shaking tables and magnetic separation techniques to upgrade Ta_2_O_5_ and Nb_2_O_5_ from Songzi, China, achieving final grades of 8.96% Ta_2_O_5_ and 5.43% Nb_2_O_5_^9^.

Our research group investigated the upgrading of tantalum and niobium from Abu Dabbab tantalite ore, of particle size of + 75 microns, using a shaking table concentrator and dry magnetic separation. The beneficiation process achieved an enrichment ratio of up to 160-fold for both Ta_2_O_5_ and Nb_2_O_5_. Acid leaching reduced thorium oxide (ThO_2_) by 73% and uranium oxide (U_3_O_8_) by 68%. Aliquat 336 ionic liquid was applied to separate Ta_2_O_5_ with 90% purity, while Amberlite anion exchanger with 6.0 M HCl was used to isolate Nb_2_O_5_ with 87% purity^2^. Shaking tables are limited to processing coarse particles (+ 75 microns) and are not effective for fine materials. To enhance the beneficiation efficiency for fine particles, centrifugal concentrators are utilized^10,11^.

Nzeh et al. reported numerous studies on tantalite separation that combined physical and chemical separation techniques with detailed process optimization. They examined the grindability and work index of alluvial columbite to estimate energy consumption costs. Their study on alkaline roasting demonstrated the effective dissolution of niobium and tantalum into a leach solution, avoiding the use of harmful hydrofluoric acid. Additionally, they reported heavy minerals upgrading, including columbite-tantalite, by implementing a two-stage separation: initial gravity-based techniques such as jigging tabling, and hydrocycloning, followed by magnetic separation or flotation, enhancing tantalum recovery. They concluded that advanced gravity concentration notably improves the separation efficiency of fine particles. Nzeh et al. recommended further research into comprehensive mineral characterization and process optimization to achieve sustainable tantalum processing^1,12–18^.

The Falcon SB40 enhanced gravity concentrator is designed to recover fine minerals and operates at higher gravitational forces, up to 300 Gs. It has been successfully used to upgrade fine, low-grade cassiterite ore^19^. The Falcon concentrator has also been employed in upgrading oil shale to recover kerogen as an alternative energy source^20^. It has also been used successfully to upgrade ilmenite from the Abu Ghouson deposits of Egypt^21^. Marion et al. (2017) explored the potential of dense medium separation of mineral fines using Falcon concentrator and found that both lab centrifuges and modified Falcon concentrators delivered similar performances when processing fine rare earth minerals^22^. Aydogan and Kademli (2019) evaluated the effects of particle size distribution on Falcon efficiency which was improved with narrower size fractions^23^. Nete et al. (2014) employed magnetic separation techniques to upgrade Nb_2_O_5_ and Ta_2_O_5_^24^.

However, gaps remain in optimizing multi-stage separation for ultra-fine, low-grade ores and integrating statistical design to enhance efficiency and this study would address these challenges. Therefore, the objective of this study is to develop a scalable, efficient, and sustainable process for recovering tantalum and niobium from ultra-fine (-75 μm) low-grade Abu Dabbab tantalite ore. By optimizing physical separation techniques—including the Falcon SB40 concentrator and Boxmag magnetic separator—and employing a Box-Behnken design to refine parameters, high enrichment ratios of Ta_2_O_5_ and Nb_2_O_5_ would be achieved. Additionally, the process seeks to valorize secondary minerals (cassiterite, albite, and mica), thereby addressing key technical, efficiency, and sustainability challenges in Ta-Nb beneficiation.

## Materials and methods

### Materials

The samples were gathered from the Abu Dabbab area, situated 20 km north of Mersa Alam city, at coordinates 25º20’ 27’’ N latitude and 34º 32’ 30’’ E longitude. The Abu Dabbab tantalite deposit is geologically complex, containing albite-rich apogranites with disseminated tantalite, columbite, and cassiterite. 30 bulk samples (10 kg each) were collected from three distinct zones; central albite-tantalite, peripheral quartz-mica, and transitional following a grid-based. Sampling followed a grid-based approach and was analyzed via XRF. The principal component analysis ensured representativeness, validating the sampling method with no significant deviation (*p* > 0.05) from the Egyptian Geological Survey data^2,6^. Box-Behnken Design Software: The experimental design was performed using a software package, Design-Expert 12.0.3, Stat-Ease, Inc., Minneapolis, USA, for regression analysis of experimental data and to plot the response surface^25^.

### Abu Dabbab ore sample preparation

The collected ore samples were mixed together as one representative sample, crushed using a jaw crusher, and the resulting material was then ground in a rod mill to achieve the appropriate size (−75 microns) for processing in the Falcon SB40 concentrator. The initial chemical composition of the Falcon feed (Table 1) included 0.025% Ta_2_O_5_ and 0.009% Nb_2_O_5_, as confirmed by XRF analysis. The particle size distribution (PSD) of the Falcon feed was analyzed using Malvern Mastersizer 2000, showing d_10_ = 11.21 μm, d_50_ = 36.42 μm, and d_90_ = 68.15 μm.

**Table 1:** Chemical analysis of the original tantalite ore sample.

Wt., % of tantalite ore components											
SiO_2_	Al_2_O_3_	Na_2_O	K_2_O	CaO	TiO_2_	MnO	Fe_2_O_3_	Nb_2_O_5_	SnO_2_	Ta_2_O_5_	L.O.I
55.07	26.36	11.55	2.99	0.235	0.122	1.28	0.671	0.009	0.09	0.025	1.50

MgO	P_2_O_5_	SO_3_	Cr_2_O_3_	ZnO	Ga_2_O_3_	Rb_2_O	ZrO_2_	SrO	Cl	ThO_2_	U_3_O_8_
0.045	0.022	0.029	0.028	0.016	0.006	0.080	0.010	0.003	0.002	0.002	–

### Gravity separation techniques

The Falcon technique was conducted to upgrade particles in the − 75-micron size.

#### Falcon SB40 experiments

Tantalum beneficiation tests were conducted using a Falcon SB40 centrifugal concentrator. The prepared feed material, d_80_ = 52 μm, introduced as a slurry of 20.0% solid-to-liquid ratio through a central vertical feed pipe, was propelled by an impeller. As the particles were moved up the sloping, rubber-lined rotor wall (migration or segregation zone), rapid stratification occurred according to specific gravity within a high gravity field. No water was injected in this area. In the retention or separation zone, located just above the migration zone, fluidized water was inserted through the rotor wall to establish a fluidized bed. Dense particles were retained in this zone until the falcon was stopped, at which point the concentrate was driven out. Three variables were optimized; Feed rate (80–120 g/min), Fluidization water (2.0–6.0 psi) and Centrifugal field (150–250 G’s). To achieve high-grade concentrates, the produced pre-concentrates were cleaned twice. Both concentrates and tailings were collected, dried, weighed, and subjected to chemical analysis^19–21^.

### Magnetic separation

Wet high-gradient magnetic separation experiments were performed using a “Boxmag Rapid” LHW magnetic separator. The separating canister, filled with stainless steel wool (specific gravity of 7.13 g/cm³), has a rectangular shape with dimensions of 37 mm in width, 82 mm in length, and 190 mm in height, resulting in a filling volume of 600 mm³. The tests were carried out under predetermined optimal conditions: a matrix wire diameter of 300 μm and a retention time of 150 s. To prevent agglomeration, the feed was conditioned, and the dispersed sample was subjected to magnetic separation using a peristaltic pump. The slurry was gradually passed through the wet magnetic separator; the magnetic portion was adhered to the grid (stainless steel wool), while the non-magnetic portion was collected at the bottom. The magnetic portion was removed from the separating grid by washing with water in the absence of a magnetic field. Three parameters were studied: field intensity (1.5–2.0 tesla), matrix loading capacity (ratio of the volume of stainless-steel wool to the canister volume) (15–25%), and feed pulp density (solid-to-liquid ratio) (8.0–16.0%). The magnetic field intensity was adjusted via controlling the input current intensity (ampere) and clarified magnetic field calibration using High Precision TESLA METER TM-901EXP (± 0.01 T accuracy). Both magnetic and non-magnetic fractions were weighed and chemically analyzed^19^.

### Instrumentation

Quantitative chemical analysis of the oxide content in the original and processed tantalite samples was performed using an X-ray fluorescence (XRF) spectrometer, model WD Axios Advanced (Panalytical, Netherlands). The instrument, which employs SuperQ software for standard-based analysis, was used to detect the major oxides and trace elements present in the samples. To prepare the samples, 10 g was mixed with 2 g of wax as a binder, pressed into a disk within an aluminum cup, and then exposed to X-rays. For trace elements, the detection limit using this WD-XRF method is 0.01%, with an estimated error margin of ± 0.004%. Qualitative phase analysis of the minerals was carried out using X-ray diffraction (XRD). The analysis was performed with a PANalytical X’Pert PRO diffractometer, which features a secondary monochromator, using Cu Kα radiation (λ = 1.542 Å), working at 45 kV and 35 mA, with a scanning speed of 0.04°/sec. The diffraction data were collected over a 2θ range of 2° to 80°, yielding information on interplanar spacing (d, Å) and relative intensities (I/Io). The peak intensities in the diffraction pattern correspond to the atomic distribution within the crystalline lattice. The resulting XRD patterns were compared to the ICDD database for phase identification. Elemental analysis of the samples was conducted using a Shimadzu ICPS-7510 ICP-OES, a sequential plasma spectrometer. A Nikon reflected polarized microscope was utilized to examine the optical features and petrography of tantalite. Photomicrographs of tantalite samples were captured with a 1600 × 8 MP LED digital electronic microscope. The magnetic susceptibility of both original tantalite and produced fractions was determined using a Bartington MS3 susceptibility meter (UK) equipped with an MS2G sensor for powder samples. Data was displayed and stored on a computer connected to the meter. Each sample was placed in a 1 cm³ plastic vial for measurement, following calibration with a standard provided by the manufacturer. The MS2G meter operates with a magnetic field strength of 500 µT and a frequency of 1.3 kHz. Three measurements were taken for each sample, and the average value is presented in this paper. Results are reported as dimensionless values in the cgs unit system^26,27^.

## Results and discussion

### Characterization of the tantalite sample

**Fig. 1:**
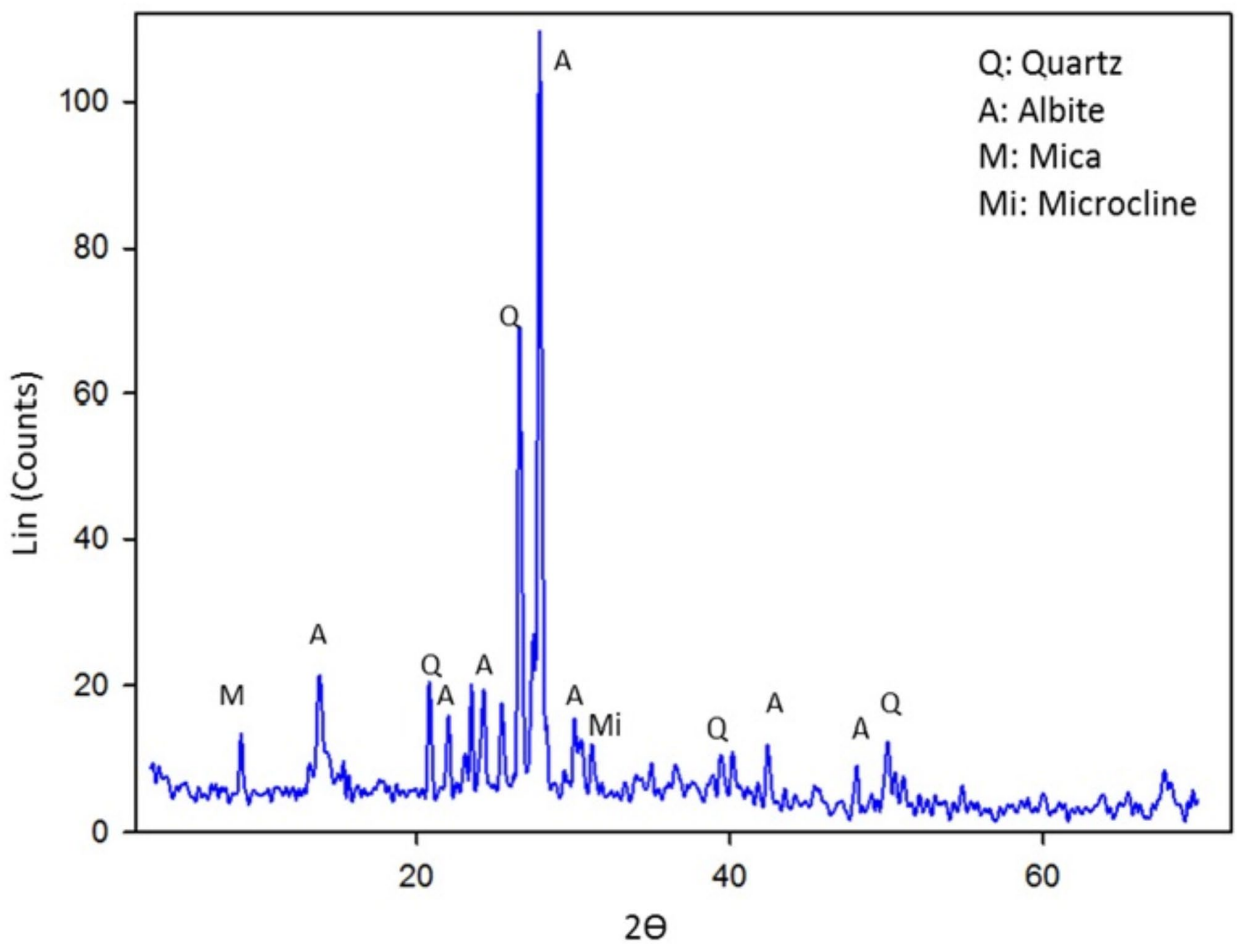
XRD of the original tantalite ore sample.

The X-ray diffraction (XRD) pattern of the original tantalite feed sample reveals the presence of many mineral phases, primarily albite, quartz, and muscovite. Silicates constitute the primary composition of the sample, with a low concentration of metallic minerals indicating a heterogeneous and complex mineralogical composition (Fig. 1).

A comprehensive chemical analysis of the original tantalite reveals its composition to be approximately 55% SiO_2_, 26% Al_2_O_3_, 11% Na_2_O, and 3% K_2_O, with trace amounts of tin, tantalum, niobium oxides (Table 1). Petrographic analysis under transmitted light reveals that the tantalite falcon feed sample has a granitic composition, predominantly consisting of plagioclase (mainly albite), quartz, and muscovite. Dark opaque minerals, including cassiterite and tantalite, are present as irregular masses embedded within a silicate gangue (Fig. 10A, B). Photomicrographs of the feed sample (Fig. 11A) display a heterogeneous texture, characterized by a significant presence of albite.

**Fig. 2:**
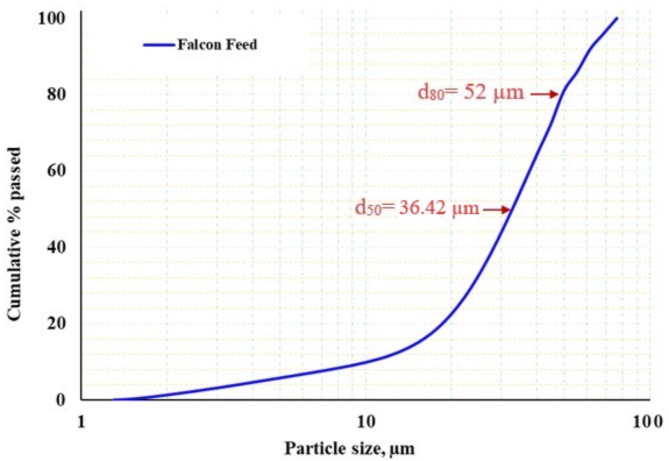
Particle size distribution of the Falcon feed.

The particle size distribution of the tantalite feed sample is presented in (Fig. 2). The particle size distribution (PSD) of the Falcon feed showed d_10_ = 11.21 μm, d_50_ = 36.42 μm, and d_90_ = 68.15 μm. The “span value,” defined as (d_90_-d_10_)/d_50_, is a useful metric for assessing the width of the particle size distribution (PSD). A decrease in this value indicates a narrower PSD, reflecting greater uniformity in particle sizes^28^. Additionally, the Steepness Factor (SF = d_50_/d_20_) is used to classify distributions, with values above 2 indicating a broad distribution and values below 2 representing a narrow one, which is often preferred in many applications^29^. Table 2 presents the characteristics of the Falcon feed distribution, which is notably narrow and uniform. This uniformity allows the Falcon device to separate particles primarily based on density rather than size.

**Table 2:** Particle size distribution width values for Falcon feed.

PSD characteristics	d_10_	d_20_	d_50_	d_90_	SF (d_50_/d_20_)	Span (d_90_-d_10_)/d_50_
Falcon feed	11.21	21.12	36.42	68.15	**1.72**	**1.56**

### Ore beneficiation

The effective upgrading of tantalum and niobium minerals primarily relies on the physicochemical features of the ore, including differences in the specific gravity between tantalum, niobium, and associated minerals, magnetic susceptibility, the presence of radioactive elements, and the tantalum and niobium content, which all relate to the ore’s nature. For low-grade tantalite ore with Ta_2_O_5_ and Nb_2_O_5_ contents below 0.1%, the beneficiation process starts with an enrichment step. Gravity separation techniques utilize Earth’s natural gravitational force (1G) to separate heavier tantalum and niobium minerals from lighter silicate minerals. Enhanced gravity equipment further separates the heavy and light fractions using centrifugal forces up to 300 G. The magnetic separation can then be used to enrich Ta_2_O_5_ and Nb_2_O_5_^24^.

#### Beneficiation using the gravity separation technique

Given the substantial difference in specific gravity between valuable minerals (tantalite with a specific gravity of 7.1 and columbite with 5.2) and gangue silicates (2.6), gravity separation is recommended^7,21^. In this study, the Falcon SB40 concentrator was used to enrich fine, low-grade tantalite ore with a feed size of -0.075 mm.

##### Application of the Box-Behnken design

The Box-Behnken design technique was employed to optimize beneficiation using the Falcon SB40 concentrator. This approach allowed for the assessment of how various factors, feed rate, fluidization water, and centrifugal field, affect the process and interact with one another. The Falcon SB40 concentrator factor levels and design results are given in Appendix A (Tables A1, A2).

Optimal conditions were determined using a second-order polynomial function that established a relationship between the investigated variables and their responses. The following equation represents the general form^25–27^:

1 $$Y\,=\,{\beta _{\text{o}}}\,+\,{\beta _{\text{1}}}{X_{\text{1}}}\,+\,{\beta _{\text{2}}}{X_{\text{2}}}\,+\,{\beta _{\text{3}}}{X_{\text{3}}}\,+\,{\beta _{{\text{12}}}}{X_{\text{1}}}{X_{\text{2}}}\,+\,{\beta _{{\text{13}}}}{X_{\text{1}}}{X_{\text{3}}}\,+\,{\beta _{{\text{23}}}}{X_{\text{2}}}{X_{\text{3}}}\,+\,{\beta _{{\text{11}}}}{X_{\text{1}}}^{{\text{2}}}\,+\,{\beta _{{\text{22}}}}{X_{\text{2}}}^{{\text{2}}}\,+\,{\beta _{{\text{33}}}}{X_{\text{3}}}^{{\text{2}}}$$

Where *Y* is the expected response; tantalum oxide grade and recovery %, *X*_1_, *X*_2_ and *X*_3_ are the studied parameters: feed rate, fluidization water, and centrifugal field; *β*_*ij*_ are the constants and coefficients in the equation.

The analysis of variance (ANOVA) was employed to assess the statistical parameters. The coefficient of determination, R^2^, was used to determine how well the experimental results fit the polynomial model. The relevance of each term in the equation was assessed using an F-test, while adequate precision was used to quantify the signal-to-noise ratio, with a value exceeding 4 indicating a suitable signal^25–27^. The ANOVA results for the tantalum beneficiation system confirm the accuracy of the polynomial model, as evidenced by model F-values of 76.78 and 110.63 and adequate precision ratios of 22.36 and 37.46 (Table 3). The Ta_2_O_5_ grade and recovery percentages can be calculated using Eqs. (2) and (3) derived from the design:

2 $$\begin{gathered} {\mathbf{T}}{{\mathbf{a}}_{\mathbf{2}}}{{\mathbf{O}}_{\mathbf{5}}}\% = \hfill \\ +\,{\text{1}}.{\text{9}}0\, - \,0.{\text{25}} \times {\text{A}}\,+\,0.{\text{24}} \times {\text{B}} - \,0.{\text{16}} \times {\text{C}} - \,0.{\text{25}} \times {{\text{A}}^{\text{2}}} - \,0.{\text{3}}0 \times {{\text{B}}^{\text{2}}} - \,0.{\text{35}} \times {{\text{C}}^{\text{2}}} - \,0.0{\text{2}} \times {\text{A}} \times {\text{B}}\,+\,0.0{\text{8}} \times {\text{A}} \times {\text{C}} - \,0.0{\text{2}} \times {\text{B}} \times {\text{C}} \hfill \\ \end{gathered}$$

3 $$\begin{gathered} {\mathbf{T}}{{\mathbf{a}}_{\mathbf{2}}}{{\mathbf{O}}_{\mathbf{5}}}\;{\mathbf{Recovery}}{\text{ }}\% = \hfill \\ +\,{\text{85}}.{\text{92}}\, - \,0.{\text{3}}0 \times {\text{A}}--{\text{1}}.{\text{37}} \times {\text{B}}\,+\,{\text{2}}0.0{\text{5}} \times {\text{C}} - \,0.{\text{42}} \times {{\text{A}}^{\text{2}}} - \,0.{\text{77}} \times {{\text{B}}^{\text{2}}} - \,0.{\text{77}} \times {{\text{C}}^{\text{2}}} - \,0.{\text{38}} \times {\text{A}} \times {\text{B}} - \,0.{\text{17}} \times {\text{A}} \times {\text{C}} - 0.{\text{13}} \times {\text{B}} \times {\text{C}} \hfill \\ \end{gathered}$$

**Table 3:** ANOVA for response surface quadratic model of Falcon concentrator.

The statistical parameters	Falcon SB40	
	Grade, %	Recovery, %
The standard deviation	0.060	0.240
R-squared	0.990	0.993
Adequate precision	22.36	37.46
The model F-values	76.78	110.63

Where: A is Feed rate (g/min), B is Fluidization water (psi) and C is Centrifugal field (G’s).

**Fig. 3:**
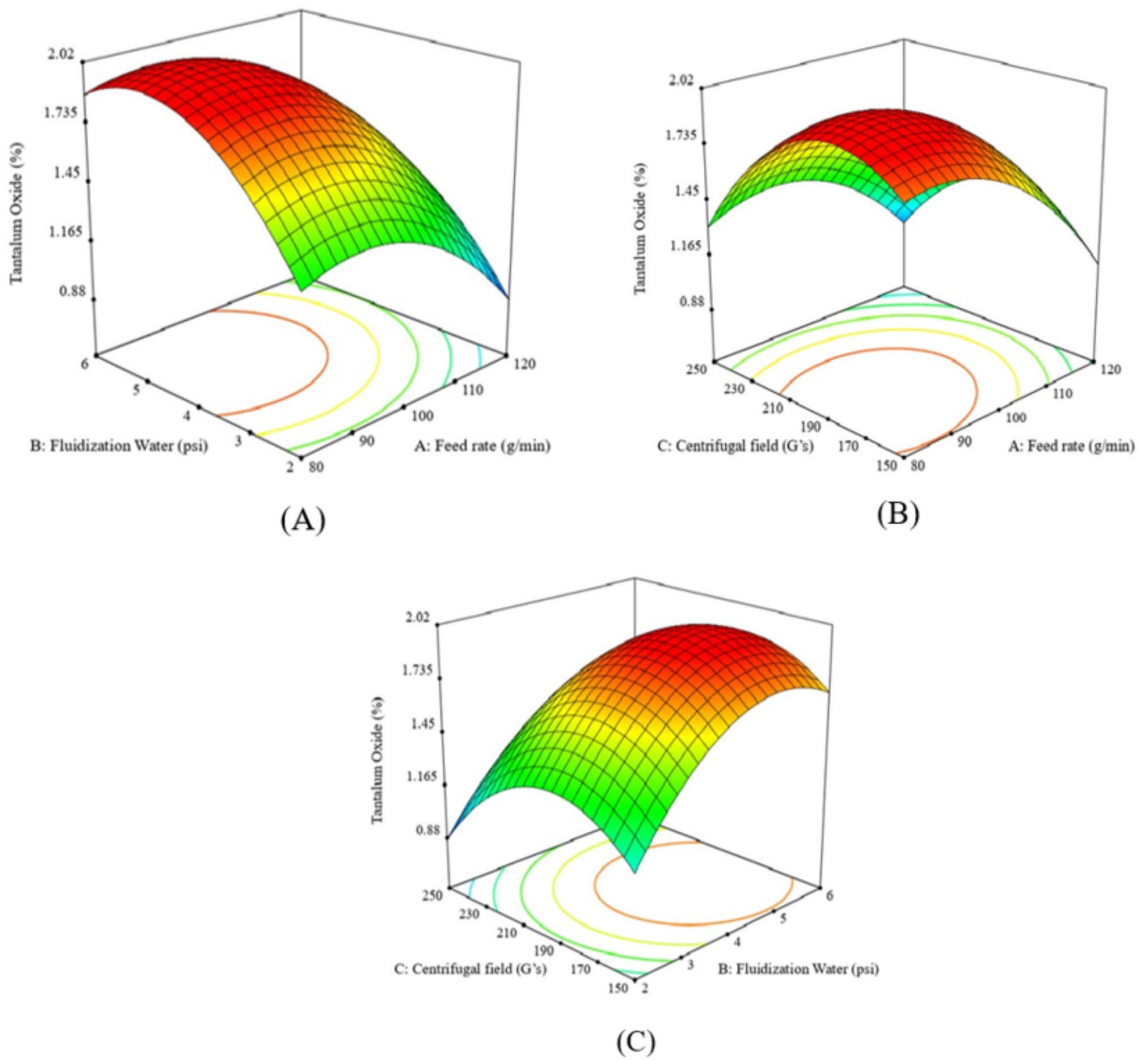
The response surface plots (**A**–**C**) of Ta_2_O_5_% resulting from the main effects of falcon SB40 concentrator variables; feed rate, fluidization water and centrifugal field.

A Ta_2_O_5_ grade of 1.90% was achieved at a fluidization water pressure of 4.40 psi and a centrifugal field of 200 G’s. Higher water pressure and centrifugal field values resulted in a decrease in tantalum grade (Fig. 3A–C). The Falcon bowl features a restricted fluidized groove surface at the top circular section and a larger non-fluidized conical section at the bottom. Lower fluidization water levels were insufficient to separate lighter particles (albite) from heavier particles (tantalite), resulting in lower tantalum grades. Consequently, the Falcon system typically needs a higher volume of fluidization water. In the retention zone, the fluidization water helps establish a fluidized bed, and increasing its flow enhances the Ta_2_O_5_ grade. However, exceeding the gravitational forces beyond 200 G’s resulted in a decrease of Ta_2_O_5_ grade, as low dense albite particles could be retained with the heavier tantalite particles at very high G’s. High tantalite recoveries, up to 88.0%, were achieved at high centrifugal fields with low feed rates and water pressure levels. Increasing the feed rate and water pressure reduced recovery, (Fig. 3A–C). These findings align with those reported by Nzeh et al., (2023) as excessive feed rates hinder stratification, while very high G-forces may risk particle entrainment^1^.

**Fig. 4:**
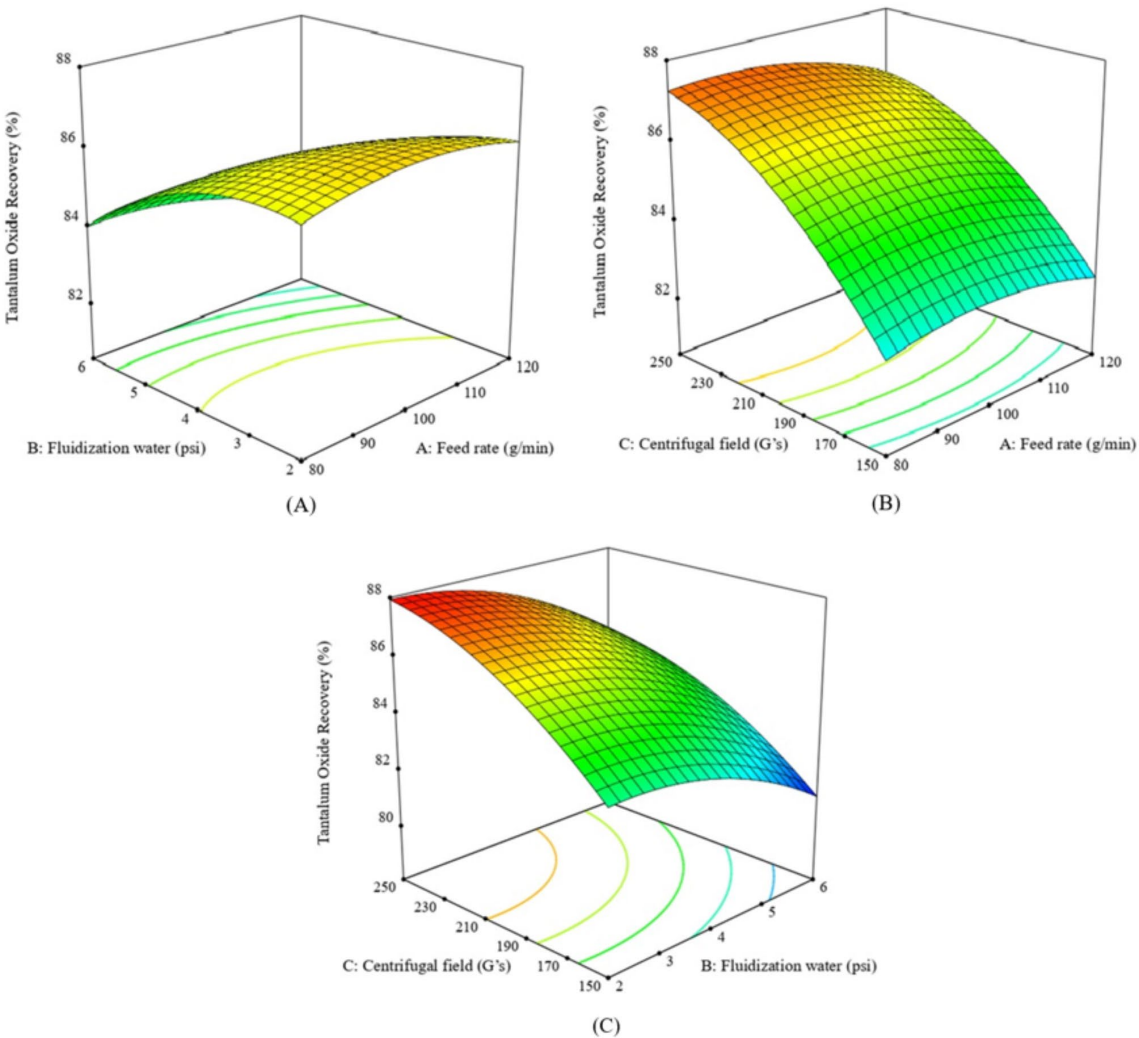
The response surface plots (**A**–**C**) of Ta_2_O_5_ recovery % resulting from the main effects of falcon SB40 concentrator variables; feed rate, fluidization water and centrifugal field.

At moderate gravitational forces (150 G’s), lower Ta_2_O_5_ recovery was observed even with higher water pressures, likely because the rotation velocities of Falcon SB40 were insufficient to retain dense tantalite particles. rising the gravitational force up to 250 G’s improved recovery. High G’s were better for retaining dense tantalite particles, but the recovery decreased when the water pressure exceeded 4 psi, as some dense tantalite particles were lost to the zone of lighter particles (Fig. 4A–C). High Ta_2_O_5_ grade and recovery were achieved at lower feed rates. Increasing feed rates affected particle stratification and decreased the upgrading efficiency of albite from tantalite particles, (Figs. 3 and 4A–C).

**Fig. 5:**
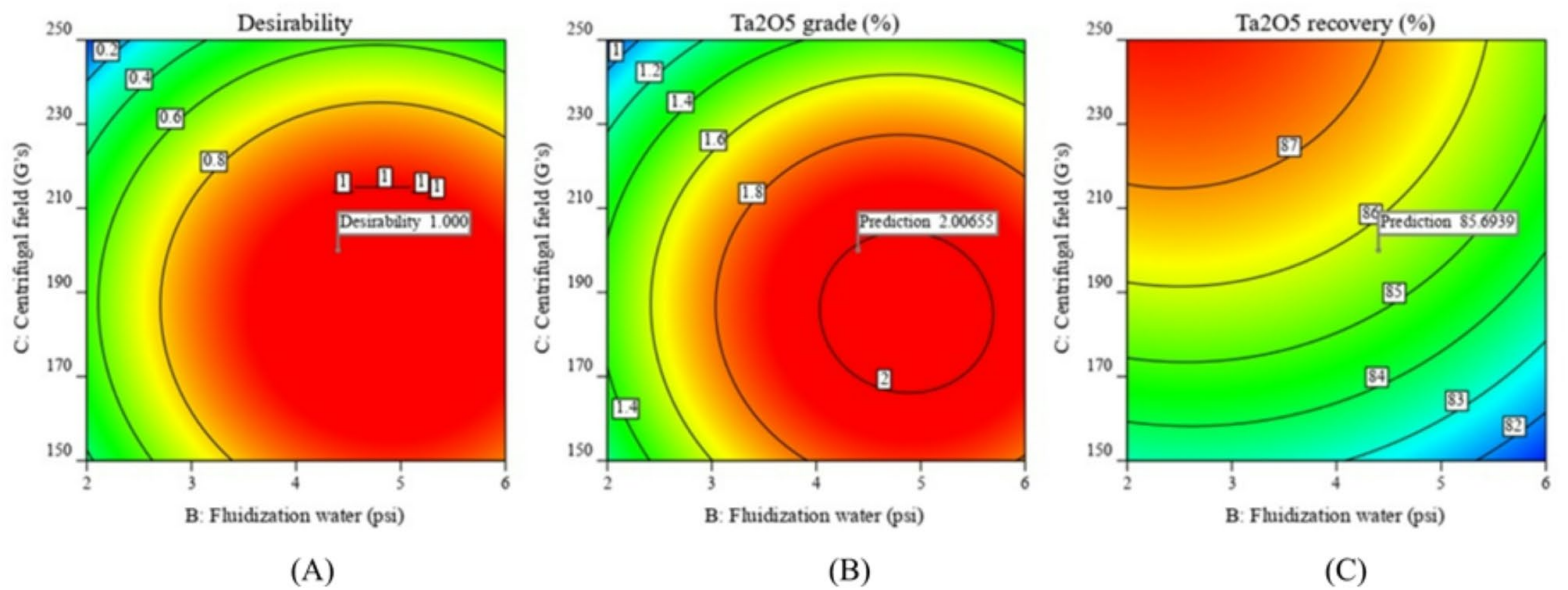
Optimization of the tantalite beneficiation; (**A**) Desirability, (**B**) Ta_2_O_5_ grade % and (**C**) Ta_2_O_5_ recovery % using falcon SB40 concentrator.

The optimal variables for tantalum beneficiation using the Falcon SB40 separator, as determined by the Box-Behnken design, are a feed rate of 89.30 g/min, fluidization water at 4.40 psi, and a centrifugal field of 200.0 G’s (Table 4). With these variables, a concentrate of 2.00% Ta_2_O_5_ and 0.705% Nb_2_O_5_ was obtained, with operational recoveries of 85.70% and 84.35%, respectively (Fig. 5A–C).

**Table 4:** Optimum parameters for tantalite beneficiation using Falcon SB40 concentrator.

Falcon SB40 concentrator	
Feed rate (g/min)	89.30
Fluidization water (psi)	4.40
Centrifugal field (G’s)	200.00

#### Tantalite enrichment using magnetic separation

Magnetic separation was applied to the Falcon-concentrated tantalite to enhance the enrichment ratio. The Box-Mag Rapid LHW magnetic separator, a high-gradient wet magnetic separator, was used, and parameters such as field intensity, matrix loading capacity, and feed pulp density were studied.

##### Application of the Box-Behnken design

The Box-Behnken Design was applied for optimizing the enrichment of tantalum using the Box-Mag Rapid LHW magnetic separator and to assess interactions between different parameters. The Box-Mag magnetic separator factor levels and design results are given in Appendix B (Tables B1, B2).

The optimized factors were evaluated using a second-order polynomial function that generated a relation between the investigated variables and response using Eq. 1, where *Y* is tantalum oxide grade and recovery %, *X*_1_, *X*_2_ and *X*_3_ are studied parameters: field intensity, matrix loading capacity, and feed pulp density; *β*_*ij*_ are the constants and coefficients in the equation.

ANOVA results for the tantalum beneficiation system confirmed the fit of the results of experiments to the polynomial equation, validating its accuracy (Table 5). The model F-values of 687.21 and 180.28 indicate the significance of the model, and adequate precision ratios of 93.06 and 45.34 indicate a strong signal. The Ta_2_O_5_ grade and recovery could be assumed using Eq. (4) and Eq. (5) derived from the design^25–27^:

4 $$\begin{gathered} {\mathbf{T}}{{\mathbf{a}}_{\mathbf{2}}}{{\mathbf{O}}_{\mathbf{5}}}\% = \hfill \\ +\,{\text{5}}.{\text{55}}\,+\,0.{\text{8}}0*{\text{A}}\,+\,0.{\text{18}}*{\text{B}} - \,0.{\text{2}}0*{\text{C}} - \,0.{\text{16}}*{{\text{A}}^{\text{2}}} - \,0.0{\text{6}}*{{\text{B}}^{\text{2}}} - \,0.{\text{34}}*{{\text{C}}^{\text{2}}} - \,0.0{\text{5}}*{\text{A}}*{\text{B}}\,+\,0.0{\text{9}}*{\text{A}}*{\text{C}} - \,0.0{\text{5}}*{\text{B}}*{\text{C}} \hfill \\ \end{gathered}$$

5 $$\begin{gathered} {\mathbf{T}}{{\mathbf{a}}_{\mathbf{2}}}{{\mathbf{O}}_{\mathbf{5}}}\;{\mathbf{Recovery}}{\text{ }}\% = \hfill \\ +\,{\text{94}}.{\text{45}}\,+\,0.{\text{41}}*{\text{A}}--0.{\text{37}}*{\text{B}} - 0.{\text{99}}*{\text{C}} - \,0.0{\text{9}}*{{\text{A}}^{\text{2}}} - \,0.{\text{12}}*{{\text{B}}^{\text{2}}}--{\text{1}}.{\text{25}}*{{\text{C}}^{\text{2}}} - \,0.{\text{18}}*{\text{A}}*{\text{B}}\,+\,0.{\text{1}}0*{\text{A}}*{\text{C}}\,+\,0.0{\text{8}}*{\text{B}}*{\text{C}} \hfill \\ \end{gathered}$$

**Table 5:** ANOVA for response surface quadratic model of boxmag.

The statistical parameters	Boxmag magnetic separator	
	Ta_2_O_5_ grade, %	Ta_2_O_5_ recovery, %
The standard deviation	0.032	0.100
R-squared	0.999	0.996
Adequate precision	93.06	45.34
The model F-values	687.21	180.28

Where: A is field intensity (tesla), B is matrix loading capacity (%) and C is feed pulp density (%).

**Fig. 6:**
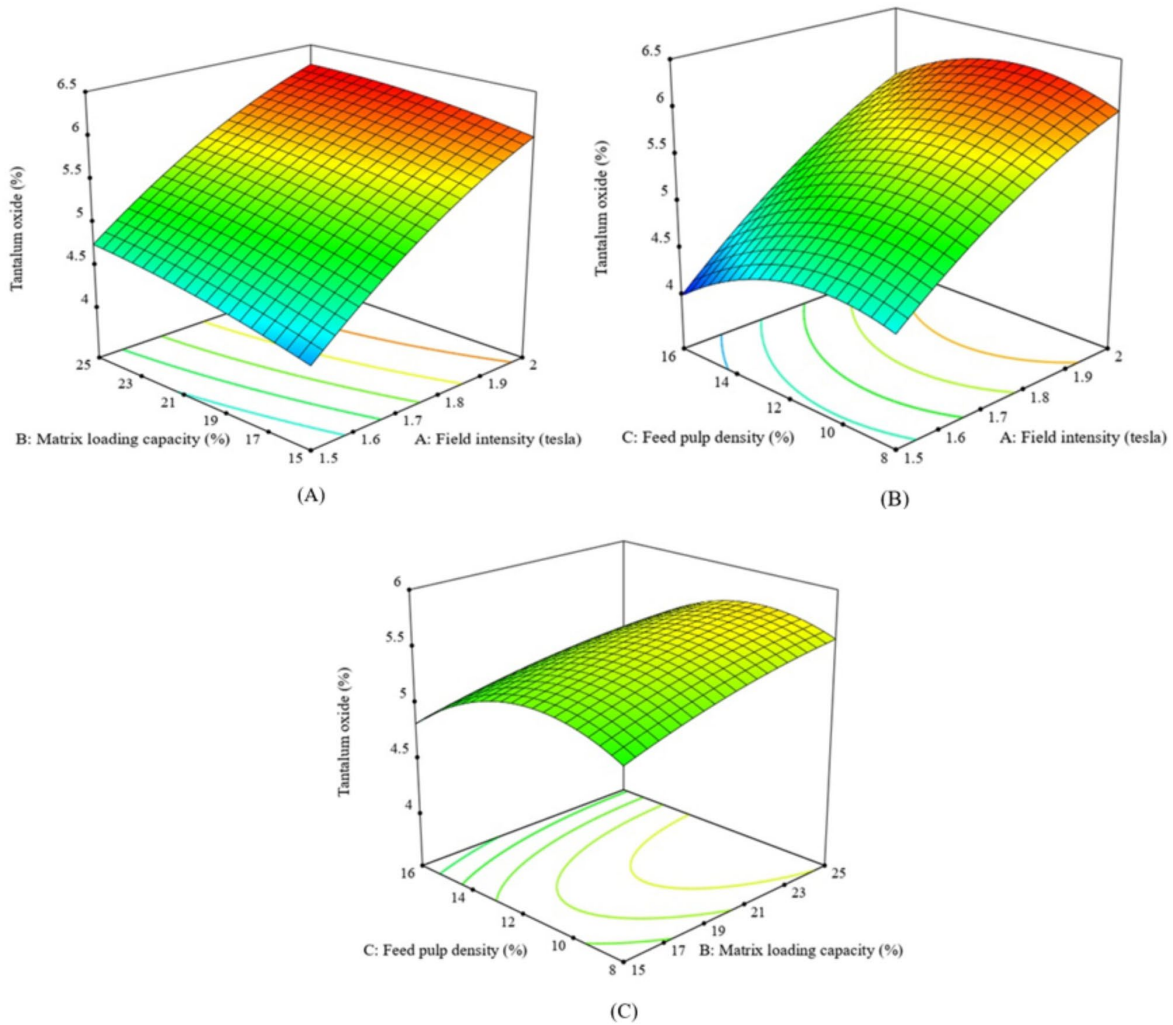
The response surface plots (**A**–**C**) of Ta_2_O_5_% resulting from the main effects of Boxmag magnetic separator variables; field intensity, matrix loading capacity and feed pulp density.

**Fig. 7:**
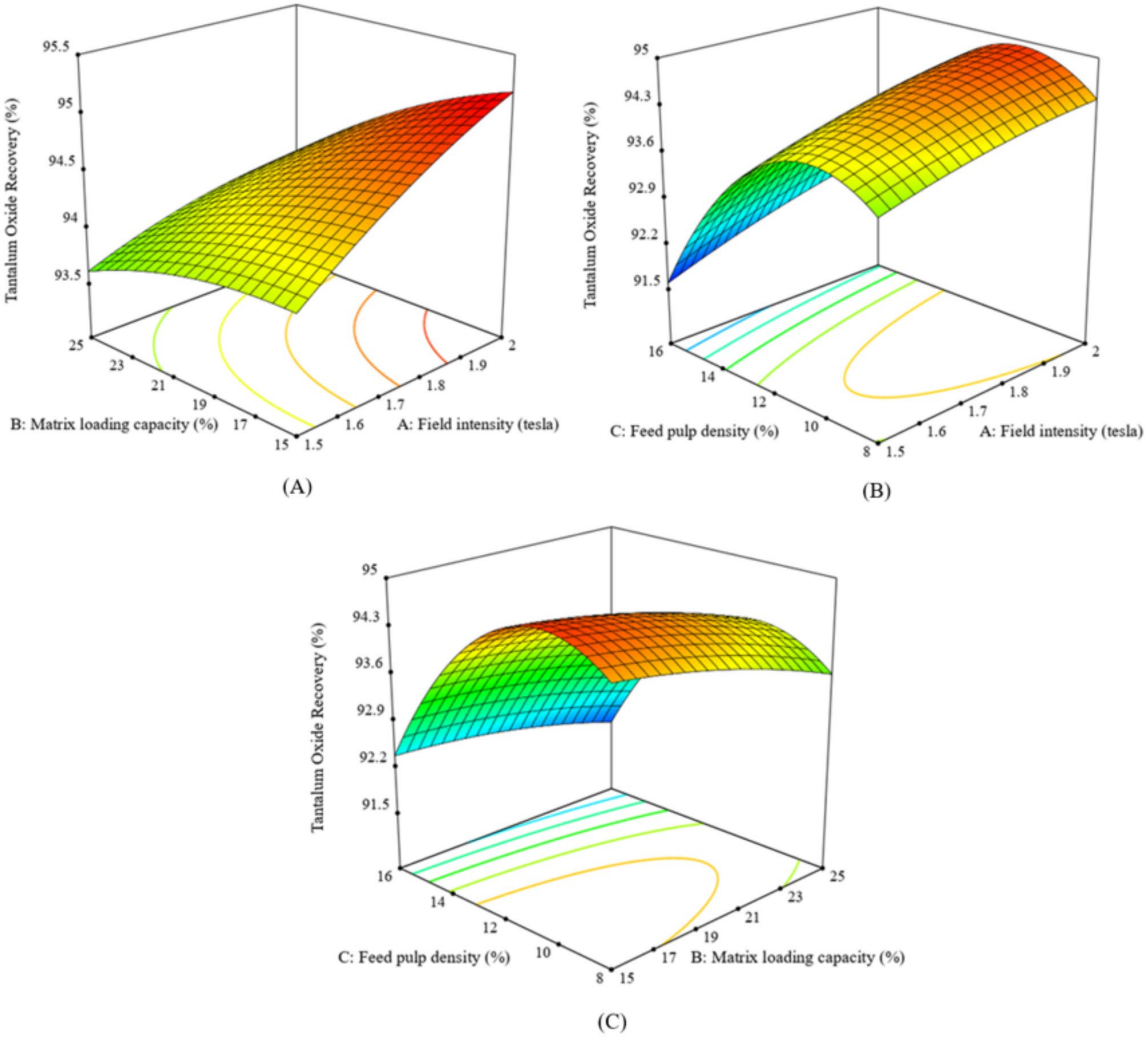
The response surface plots (**A**–**C**) of Ta_2_O_5_ recovery resulting from the main effects of Boxmag magnetic separator variables; field intensity, matrix loading capacity and feed pulp density.

**Fig. 8:**
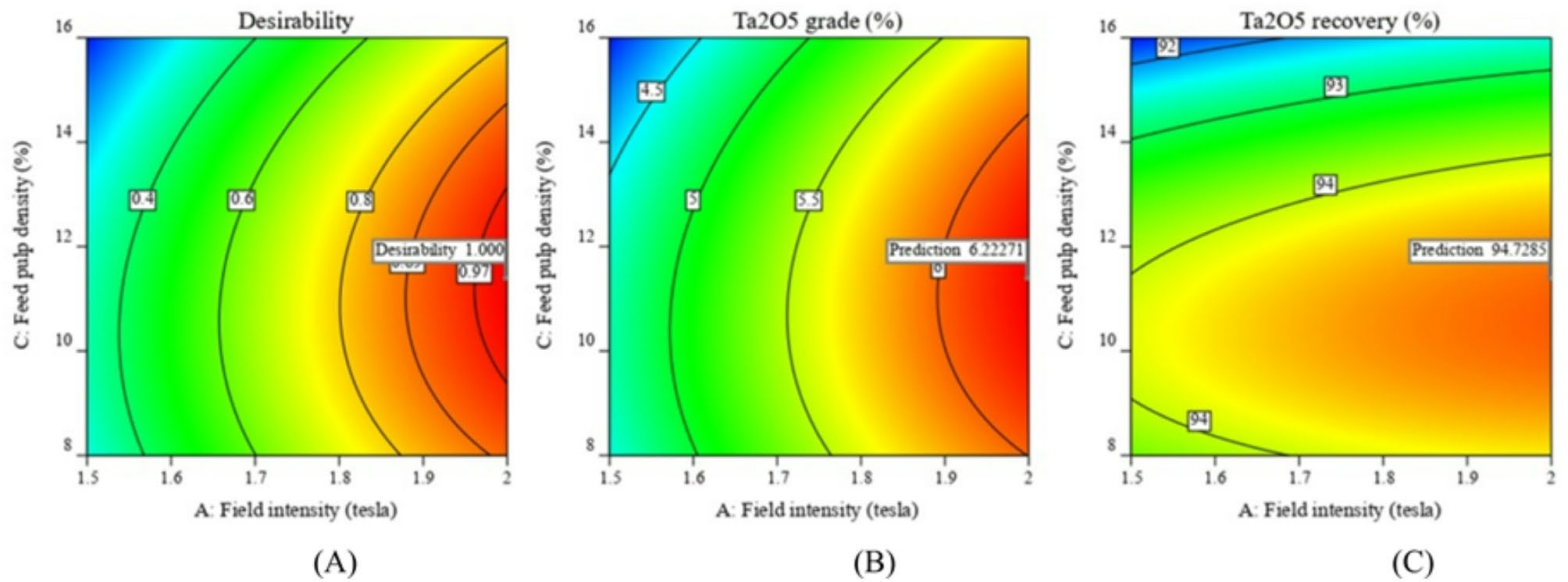
Optimization of the tantalite beneficiation; (**A**) Desirability, (**B**) Ta_2_O_5_ grade % and (**C**) Ta_2_O_5_ recovery % using Boxmag magnetic separator.

Figures 6 and 7A–C illustrate the response surfaces for magnetic tantalum concentrate grade and recovery at various levels of the studied parameters. High field intensity is crucial for separating the paramagnetic tantalite mineral from non-magnetic quartz and albite minerals. Increasing the field intensity to its maximum value (2.0 tesla) improved both Ta_2_O_5_ grade and recovery. Optimal Ta_2_O_5_ grades and recoveries were achieved at moderate feed pulp density levels. Increasing feed pulp density beyond 11.40% disrupted the distribution of magnetic and non-magnetic particles, resulting in crowding within the canister and reduced separation efficiency. Increasing the matrix loading capacity to 21.05% improved the beneficiation efficiency of both Ta_2_O_5_ grade and recovery. Values above 21.05% did not significantly impact the process. The optimal parameters for the Box-Behnken design of tantalum beneficiation using the Box-Mag Rapid LHW magnetic separator are a matrix loading capacity of 21.05%, a feed pulp density of 11.40%, and a maximum field intensity of 2.0 tesla, (Table 6). Under these conditions, a magnetic tantalum concentrate containing 6.22% Ta_2_O_5_ and 2.24% Nb_2_O_5_ was obtained, with operational recoveries of 94.73 and 95.4%, respectively, (Fig. 8A–C).

**Table 6:** Optimum parameters for tantalite beneficiation using boxmag.

Boxmag magnetic separator	
Field intensity (tesla)	2.00
Matrix loading capacity (%)	21.05
Feed pulp density (%)	11.40

By conducting the optimal variables of the Box-Behnken Design for tantalum upgrading to Abu Dabbab ore using the Falcon SB40 concentrator, followed by the Box-Mag Rapid LHW magnetic separator, a magnetic tantalum concentrate of 6.22% Ta_2_O_5_ with a tantalum enrichment ratio of approximately 250-fold was obtained (Table 7). As expected, niobium oxide was also successfully upgraded during tantalum beneficiation as a result of their similar properties. The magnetic tantalum concentrate contained 2.24% Nb_2_O_5_ with a niobium enrichment ratio of approximately 250-fold (Table 7), and both Ta_2_O_5_ and Nb_2_O_5_ were enriched with total recoveries exceeding 80%, (Table 9). Additionally, cassiterite (SnO_2_) was recovered as a heavy mineral, with the magnetic tantalum concentrate containing 17.50% SnO_2_ and an enrichment ratio of about 195-fold, with a cassiterite recovery of approximately 64%. To enhance economic viability, the optimal parameters from the Box-Behnken design for the Box-Mag magnetic separator were applied to the Falcon SB40 light fraction, which constitutes over 90% of the sample wt%. This led to the successful separation of the magnetic mica fraction from the non-magnetic albite fraction. These separations were studied by Hassan et al. (2017), yielding relatively pure fractions of both albite and mica^30^. The magnetic tantalum concentrate contains some radioactive materials, such as thorium and uranium oxides, which should be removed before dissolution and separation processes^2^.

**Table 7:** Chemical analysis of the magnetic tantalum concentrate.

Wt., % of magnetic tantalum concentrate components												
SiO_2_	Al_2_O_3_	Na_2_O	K_2_O	MnO	Fe_2_O_3_	Rb_2_O	Nb_2_O_5_	SnO_2_	Ta_2_O_5_	U_3_O_8_	ThO_2_	L.O.I
26.03	17.15	6.06	2.9	9.61	8.36	0.188	2.24	17.5	6.22	0.048	0.46	1.5

CaO	TiO_2_	MgO	P_2_O_5_	SO_3_	Cr_2_O_3_	ZnO	Ga_2_O_3_	GeO_2_	CeO_2_	La_2_O_5_	Nd_2_O_3_	Y_2_O_3_
0.115	0.088	0.035	0.054	0.014	1.38	0.01	0.011	0.008	0.009	0.006	0.002	0.001

#### Characterization of the produced valuable minerals

**Fig. 9:**
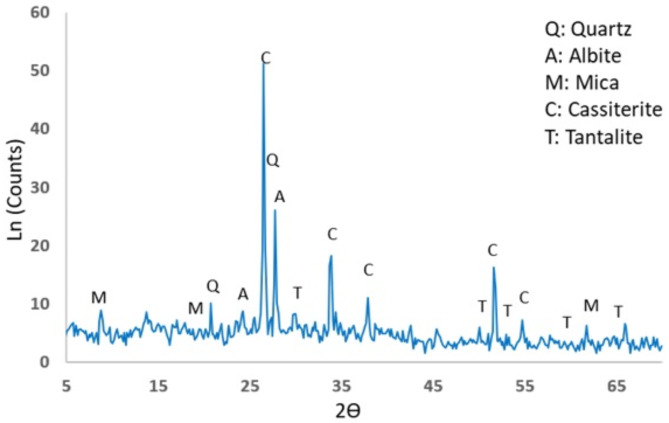
XRD of magnetic tantalum concentrate from Falcon/Boxmag.

The X-ray diffraction (XRD) pattern of the magnetic tantalum concentrate indicates the presence of tantalite and cassiterite minerals, along with silicate minerals (Fig. 9). X-ray fluorescence (XRF) analysis reveals that the concentrate consists of 6.22% Ta_2_O_5_, 2.24% Nb_2_O_5_, 17.5% SnO_2_, 26% SiO_2_, 17% Al_2_O_3_, 6% Na_2_O, 6.9% Fe_2_O_3_, 9.6% MnO, and 3% K_2_O (Table 7). Also, traces of rare earth elements (REEs), including cerium, lanthanum, yttrium, neodymium, and radioactive elements were recovered as associations to tantalum and niobium. This may refer to the recovery of monazite (Ce, La, Nd, Th) PO_4_), a phosphate mineral, which is a major source of light REEs^10^.

**Fig. 10:**
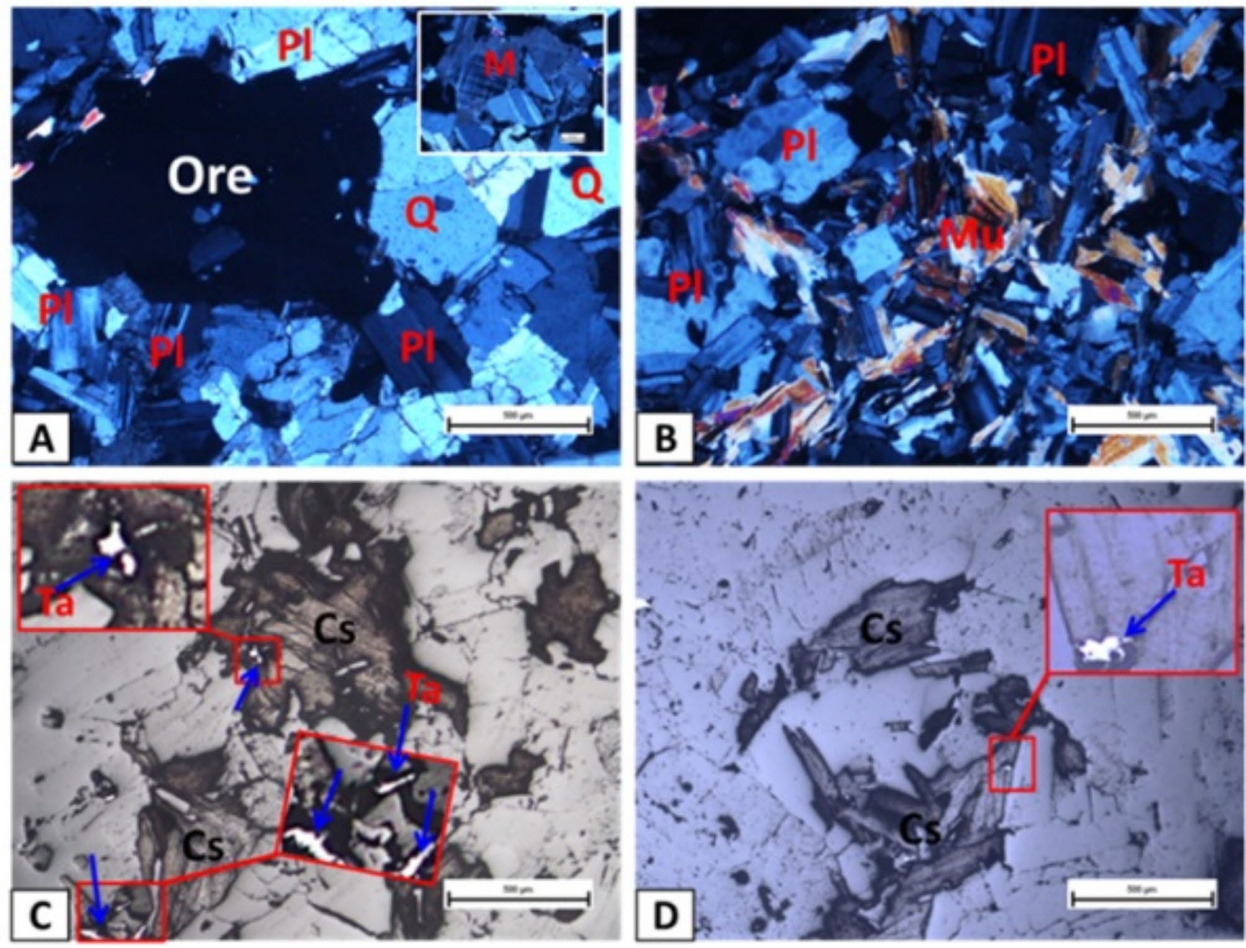
Microscopic petrography of original ore, (**A**,**B**) and heavy magnetic tantalite concentrate (**C**,**D**).

Petrographic analysis under transmitted light identifies the tantalite ore sample as granitic, primarily composed of plagioclase, quartz, microcline, and muscovite, with dark opaque minerals like cassiterite and tantalite appearing as irregular masses surrounded by silicate gangue, (Fig. 10A, B). Reflected light microscopy shows that cassiterite and tantalite exhibit a columnar habit, forming aggregates, with tantalite crystals appearing as blobs and needle-like inclusions in the magnetic tantalum concentrate, (Fig. 10C, D). These tantalite aggregates (needle-like/blobby structures) liberated from silicate gangue (albite, quartz), corroborating the high Ta_2_O_5_ recovery achieved via magnetic separation.

**Fig. 11:**
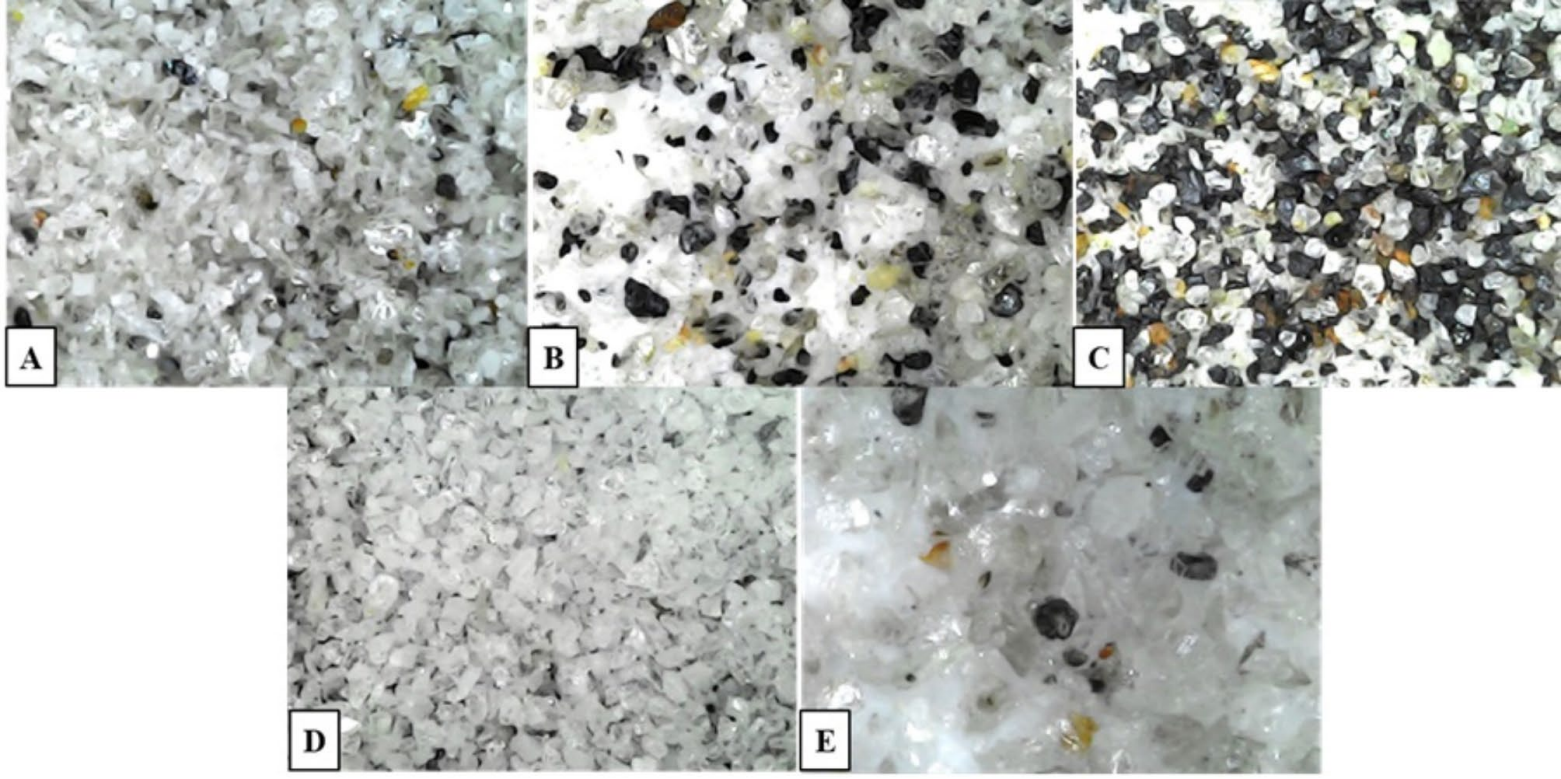
Photomicrographs captured with a 1600 × 8 MP LED digital electronic microscope, displaying the mineral composition of the: (**A**) original sample, (**B**) Falcon heavy fraction, (**C**) magnetic tantalum concentrate, (**D**) albite fraction, and (**E**) mica fraction.

Photomicrographs were used to provide a visual illustration of the effectiveness of the beneficiation process, (Fig. 11)^31^. The original sample (Fig. 11A) shows a heterogeneous mix with abundant albite, while the Falcon heavy fraction (Fig. 11B) enriches high-density minerals, making them more distinct. The Falcon heavy fraction demonstrates improved mineral density stratification, with dense cassiterite (SnO_2_) and tantalite concentrated in darker regions, aligning with the Falcon’s role in gravity separation and yielded high Ta_2_O_5_ recovery. The magnetic tantalum concentrate (Fig. 11C) shows a clear separation of tantalum minerals from gangue with high magnetic susceptibility indicated by the concentration of denser, darker particles. Cassiterite is associated with thin, black, divergent blades of tantalite-Fe. The albite fraction (Fig. 11D) contains light-white particles, reflecting its non-magnetic nature, while the mica fraction (Fig. 11E) consists of flaky structures, indicating effective separation from albite in the light falcon fraction.

The magnetic susceptibility of the achieved products is presented in (Table 8) and compared with the literature values for specific minerals^27,32^. Magnetic susceptibility measures how a material reacts to a magnetic field, which helps assess separation efficiency. The tantalite ore has a magnetic susceptibility of 1.05 × 10^−7^, indicating it is weakly magnetic. The non-magnetic light albite fraction showed a much lower susceptibility (−1.12 × 10^−8^), confirming its non-magnetic nature and aligning with literature values for the albite. The magnetic light mica fraction showed a high susceptibility (1.60 × 10^−4^), enabling its separation from albite. This value is comparable to the literature values for biotite. The magnetic heavy tantalite concentrate has a susceptibility of 1.88 × 10^−3^, indicating successful tantalite enrichment.

**Table 8:** Magnetic Susceptibility of original and treated feldspar.

Feldspar fractions (this study)	Magnetic susceptibility
Tantalite ore sample	1.05 * 10^− 7^
Non-magnetic light albite fraction	−1.12 * 10^− 8^
Magnetic light mica fraction	1.60 * 10^− 4^
Magnetic heavy tantalite concentrate	1.88 * 10^− 3^

Various minerals in literature,^27,32^	
Albite	−1.0 to −3.0 *10^− 8^
Quartz	−13 to −16.4 *10^− 6^
Biotite	1.10 * 10^− 3^
Chlorite	4.90 * 10^− 4^
Hematite	0.50–40.0 * 10^− 3^

#### Comparative analysis of tantalite upgrading from Abu-Dabbab ore and other deposits

The results in Table 9 show how both the physical upgrading techniques and the ore particle size influence the enrichment of tantalum (Ta_2_O_5_) and niobium (Nb_2_O_5_) from different low-grade ore samples. Abu-Dabbab, Egypt: The finer feed size of −75 μm used with the Falcon SB40 and Box-mag LHW during this study contributed to the high recovery of Ta_2_O_5_ (81.18%) and Nb_2_O_5_ (80.47%) with an enrichment ratio of 250 for both. Fine particle size enhances the effectiveness of these separation techniques by allowing better liberation of minerals. In contrast, a slightly coarser size (−150 + 75 μm) for the shaking table and RER magnetic separator produced a lower Ta_2_O_5_concentrate (4.48%) but a slightly higher recovery (85%), reflecting how finer particles can aid concentrate grade but may reduce recovery efficiency^2^. Songzi, China: The ore processed using gravity, magnetic separation, and grinding, at a finer size (-40 μm), achieved the highest Ta_2_O_5_ enrichment ratio (690) and a concentrate grade of 8.96%. However, the lower recovery (57.37%) suggests that while fine grinding and separation were highly effective in upgrading, they may have caused tantalum losses, possibly due to the creation of slimes or overgrinding. For Nb_2_O_5_, similar trends were observed, with moderate enrichment (494) but reduced recovery (39.55%)^9^. Larger feed sizes (D80 = 166 μm) in Jiangxi Province yielded a low Ta_2_O_5_ concentrate at 0.22% and low recovery (21.21%) due to insufficient liberation of minerals^33^. Penouta Mine, Spain: Gravity-based techniques like heavy liquid separation and shaking tables, with a feed size of -125 μm, provided moderate Ta_2_O_5_ and Nb_2_O_5_ concentrates (0.43 and 0.45%, respectively) and recoveries (63.5% for Ta_2_O_5_ and 40% for Nb_2_O_5_)^8^.

**Table 9:** Comparison between the upgrading of various low-grade tantalite ore samples.

Tantalite ore origin/reference	Upgrading techniques	Feed size, µm	Ta_2_O_5_				Nb_2_O_5_			
			Original grade %	Concentrate grade %	Recovery %	Ta-enrichment ratio	Original grade %	Concentrate grade %	Recovery %	Nb-Enrichment ratio
Abu-Dabbab – Egypt, [this work]	Falcon SB40, Box-mag LHW magnetic separator	−75	0.0252	6.22	81.18	250	0.009	2.24	80.47	250
Abu-Dabbab – Egypt,^2^	Shaking table, RER magnetic separator	−150 + 75	0.0252	4.48	85.0	178	0.009	1.61	85.0	179
Songzi – China,^9^	Gravity, magnetic separation and grinding	−40	0.013	8.964	57.37	690	0.011	5.429	39.55	494
Jiangxi Province – China,^33^	Gravity concentration: spiral chute and shaking table	−200	0.0134	0.2216	21.21	15.80	0.0069	0.1555	30.33	22.53
Penouta mine – Northwest Spain,^8^	Gravity concentration: heavy liquid separation, Mozley table, Knelson concentrator and shaking table	−125	0.0055	0.432	63.50	79.0	0.0104	0.4545	40.0	44.0

In conclusion, Particle size has a vital role in the effectiveness of upgrading techniques. The finer feed sizes in Abu-Dabbab and Songzi contributed to higher enrichment ratios but, in some cases, reduced recovery due to possible overgrinding. Coarser feed sizes, such as those used in Jiangxi, limited both recovery and enrichment due to insufficient liberation of the target minerals. Therefore, an optimal balance between particle size and upgrading techniques is essential for improving the efficiency of tantalum and niobium recovery.

## Conclusion

The beneficiation of fine-sized low-grade tantalite ore from Abu Dabbab successfully produced an enriched tantalum concentrate through a combination of gravity separation and magnetic separation techniques. The feed sample is predominantly composed of albite, quartz, and muscovite, with trace oxides (0.025% Ta_2_O_3_, 0.009% Nb₂O₅; *d*50 = 36.42 μm). The initial gravity separation was performed using the Falcon SB40 concentrator and optimized with a Box-Behnken Design. The ideal operating conditions were a feed rate of 89.30 g/min, fluidization water at 4.40 psi, and a centrifugal field of 200.0 G. Under these parameters, the concentrate achieved contained 2.00% Ta_2_O_3_ and 0.705% Nb_2_O_3_, with operational recoveries of 85.70% and 84.35%, respectively. Magnetic separation was employed to further enrich the tantalum concentrate, proving crucial in achieving higher grades and recoveries. The Box-Mag Rapid LHW magnetic separator was optimized using a Box-Behnken Design, with the ideal settings being a matrix loading capacity of 21.05%, a feed pulp density of 11.40%, and a maximum field intensity of 2.0 tesla. Under these optimal conditions, the magnetic concentrate achieved contained 6.22% Ta_2_O_3_, 2.24% Nb_2_O_3_, and 17.50% SnO_2_, along with high operational recoveries exceeding 94%. Additionally, the process produced valuable cassiterite, mica, and albite minerals, further enhancing the economic viability of the beneficiation operation. Characterization analyses confirmed the effectiveness of the separation techniques. Overall, this method offers a robust approach to upgrading fine-grained, low-grade tantalite ore, achieving enrichment ratios of up to 250 times for both Ta_2_O_3_ and Nb_2_O_3_, with total recoveries exceeding 80%.

### Recommendations

To further enhance the process, future work should begin with acid leaching to remove radioactive impurities (e.g., thorium and uranium oxides) from the magnetic concentrate. Subsequent steps involving advanced dissolution and separation methods, such as solvent extraction and ion exchange, are recommended to achieve high-purity Ta_2_O_3_ and Nb_2_O_3_. Additionally, exploring alternative separation technologies could offer further improvements in operational efficiency and concentrate quality.

## Electronic supplementary material

Below is the link to the electronic supplementary material.

Supplementary Material 1

## Data Availability

The datasets generated and/or analysed during the current study are not publicly available due to institutional roles and confidential conditions but are available from the corresponding author on reasonable request.
