# Supplementary material for: Enhanced tantalum and niobium recovery from fine-grained low-grade Abu Dabbab ore using Falcon concentration and magnetic separation
Source: Sci Rep. 2025 Mar 26;15:10432. doi: 10.1038/s41598-025-92819-7 (PMC11947099; doi:10.1038/s41598-025-92819-7)
Supplement: Supplementary file 1 — Supplementary Material 1 [file 41598_2025_92819_MOESM1_ESM.docx]

**Enhanced Tantalum and Niobium Recovery from Fine-Grained Low-Grade Abu Dabbab Ore Using Falcon Concentration and Magnetic Separation**

El-Sayed R. E. Hassan, *^1^  Yun Chen,^2^ N. A. Abdel-Khalek,^1^ and A. M. Elbendari ^1^

^1^ Minerals Beneficiation and Agglomeration Department, Minerals Technology Institute, Central Metallurgical Research & Development Institute (CMRDI), P.O. Box 87 Helwan, 11722 Cairo, Egypt.

^2^ School of Materials and Chemical Engineering, Hunan Institute of Engineering, Xiangtan, China.

(*) author to whom the correspondence should be addressed:

El-Sayed R. E. Hassan: E-mail: [prof_s_elsaidy@yhoo.com](mailto:prof_s_elsaidy@yhoo.com) - ORCID: https://orcid.org/0000-0003-4561-6696

**Appendix A**

Table A1. Falcon SB40 concentrator factor levels

| Falcon SB40 concentrator parameters | | | | | |
| --- | --- | --- | --- | --- | --- |
| Symbol | Parameter | Unit | (-) | (0) | (+) |
| A | Feed Rate | g/min | 80 | 100 | 120 |
| B | Fluidization Water | psi | 2 | 4 | 6 |
| C | Centrifugal Field | G’s | 150 | 200 | 250 |

Table A2. Box-Behnken Design results for tantalite recovery using falcon SB40 concentrator

| Run | Feed rate, g/min | Fluidization water, psi | Centrifugal field, G’s | Ta_2_O_5_ grade, % | Ta_2_O_5_ recovery, % |
| --- | --- | --- | --- | --- | --- |
| 1 | 100.00 | 4.00 | 200.00 | 1.90 | 85.90 |
| 2 | 100.00 | 4.00 | 200.00 | 1.90 | 86.00 |
| 3 | 80.00 | 6.00 | 200.00 | 1.86 | 84.00 |
| 4 | 80.00 | 4.00 | 150.00 | 1.75 | 82.90 |
| 5 | 100.00 | 2.00 | 250.00 | 0.82 | 88.00 |
| 6 | 80.00 | 2.00 | 200.00 | 1.42 | 86.20 |
| 7 | 120.00 | 4.00 | 250.00 | 1.02 | 86.20 |
| 8 | 100.00 | 4.00 | 200.00 | 1.92 | 85.80 |
| 9 | 80.00 | 4.00 | 250.00 | 1.29 | 87.00 |
| 10 | 120.00 | 4.00 | 150.00 | 1.15 | 82.80 |
| 11 | 100.00 | 4.00 | 200.00 | 1.89 | 86.00 |
| 12 | 120.00 | 2.00 | 200.00 | 0.89 | 86.20 |
| 13 | 100.00 | 4.00 | 200.00 | 1.91 | 85.90 |
| 14 | 120.00 | 6.00 | 200.00 | 1.25 | 82.50 |
| 15 | 100.00 | 6.00 | 150.00 | 1.72 | 81.00 |
| 16 | 100.00 | 6.00 | 250.00 | 1.35 | 85.20 |
| 17 | 100.00 | 2.00 | 150.00 | 1.10 | 83.30 |

**Appendix B**

Table B1. Boxmag magnetic separator Factor levels

| Boxmag rapid LHW parameters | | | | | |
| --- | --- | --- | --- | --- | --- |
| Symbol | Parameter | Unit | (-) | (0) | (+) |
| A | Field intensity | tesla | 1.50 | 1.75 | 2.00 |
| B | Matrix loading capacity | % | 15.00 | 20.00 | 25.00 |
| C | Feed pulp density | % | 8.00 | 12.00 | 16.00 |

Table B2. Box-Behnken Design results for tantalite recovery using Boxmag magnetic separator

| Run | Field intensity, tesla | Matrix loading capacity, % | Feed pulp density, % | Ta_2_O_5_ grade, % | Ta_2_O_5_ recovery, % |
| --- | --- | --- | --- | --- | --- |
| 1 | 1.50 | 15.00 | 12.00 | 4.35 | 94.0 |
| 2 | 1.50 | 25.00 | 12.00 | 4.75 | 93.7 |
| 3 | 2.00 | 20.00 | 16.00 | 5.76 | 92.6 |
| 4 | 1.50 | 20.00 | 16.00 | 3.96 | 91.5 |
| 5 | 1.75 | 25.00 | 16.00 | 5.10 | 91.8 |
| 6 | 2.00 | 20.00 | 8.00 | 5.98 | 94.5 |
| 7 | 1.75 | 25.00 | 8.00 | 5.60 | 93.5 |
| 8 | 1.75 | 20.00 | 12.00 | 5.55 | 94.5 |
| 9 | 1.75 | 20.00 | 12.00 | 5.54 | 94.4 |
| 10 | 1.75 | 20.00 | 12.00 | 5.56 | 94.5 |
| 11 | 2.00 | 15.00 | 12.00 | 6.00 | 95.1 |
| 12 | 2.00 | 25.00 | 12.00 | 6.22 | 94.1 |
| 13 | 1.75 | 20.00 | 12.00 | 5.53 | 94.4 |
| 14 | 1.75 | 15.00 | 8.00 | 5.11 | 94.5 |
| 15 | 1.50 | 20.00 | 8.00 | 4.52 | 93.8 |
| 16 | 1.75 | 20.00 | 12.00 | 5.57 | 94.4 |
| 17 | 1.75 | 15.00 | 16.00 | 4.80 | 92.5 |
